# CaMKII holoenzyme mechanisms that govern the LTP versus LTD decision

**DOI:** 10.1126/sciadv.abe2300

**Published:** 2021-04-14

**Authors:** Sarah G. Cook, Olivia R. Buonarati, Steven J. Coultrap, K. Ulrich Bayer

**Affiliations:** Department of Pharmacology, University of Colorado Anschutz Medical Campus, Aurora, CO 80045, USA.

## Abstract

Higher brain functions are thought to require synaptic frequency decoding that can lead to long-term potentiation (LTP) or depression (LTD). We show that the LTP versus LTD decision is determined by complex cross-regulation of T286 and T305/306 autophosphorylation within the 12meric CaMKII holoenzyme, which enabled molecular computation of stimulus frequency, amplitude, and duration. Both LTP and LTD require T286 phosphorylation, but T305/306 phosphorylation selectively promoted LTD. In response to excitatory LTP versus LTD stimuli, the differential T305/306 phosphorylation directed CaMKII movement to either excitatory or inhibitory synapses, thereby coordinating plasticity at both synapse types. Fast T305/306 phosphorylation required prior T286 phosphorylation and then curbed CaMKII activity by two mechanisms: (i) a cis-subunit reaction reduced both Ca^2+^ stimulation and autonomous activity and (ii) a trans-subunit reaction enabled complete activity shutdown and feed-forward inhibition of further T286 phosphorylation. These are fundamental additions to the long-studied CaMKII regulation and function in neuronal plasticity.

## Teaser

Molecular signal computation within the CaMKII holoenzyme determines the direction of synaptic plasticity.

## INTRODUCTION

**Fig. 1:**
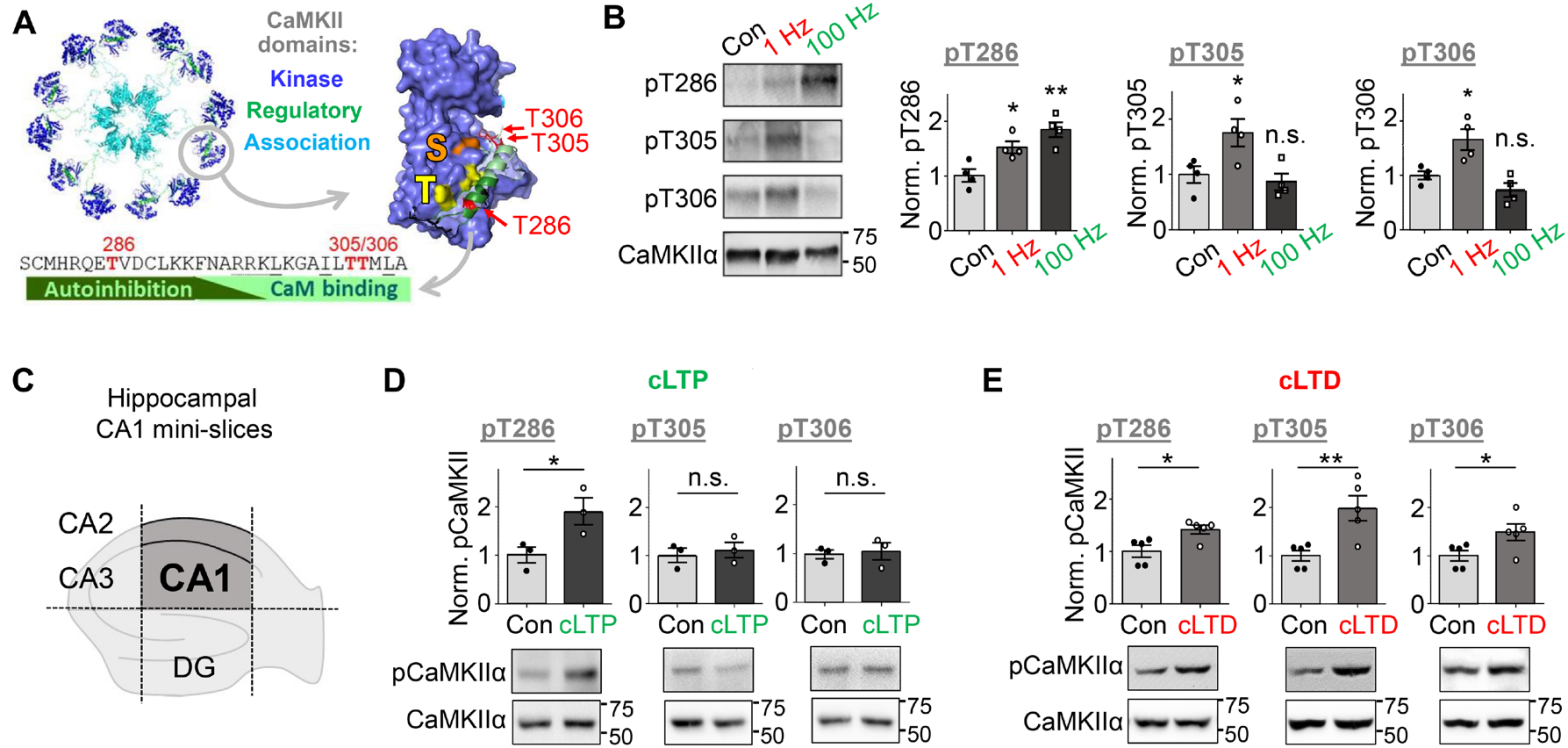
CaMKII pT305/306 is induced by LTD but not LTP stimuli. Quantifications show means ± SEM. *P < 0.05 and **P < 0.01. (**A**) Schematic of CaMKII dodecameric holoenzyme and regulatory domain sequence showing T286, T305, and T306 autophosphorylation sites within the regulatory domain. CaM binding displaces the regulatory domain to allow substrate access to the “S site” and GluN2B binding to the “T site,” which basally interacts with T286 to keep the regulatory domain in place. (**B**) Electrical stimulation of mouse hippocampal CA1 mini-slices promoted pT286 after both LTD (900 × 1 Hz) and LTP (1 s, 100 Hz) stimulation. In contrast, pT305 and pT306 was only induced by LTD stimuli (one-way ANOVA, Tukey’s post hoc test versus control, *P* = 0.0398 and 0.0025 for pT286, *P* = 0.0043 and 0.8668 for pT305, and *P* = 0.0222 and 0.4059 for pT306; *n* = 4 slices). n.s., not significant. (**C**) Schematic of hippocampal CA1 mini-slices. The dentate gyrus (DG) and the other areas of the cornu ammonis (CA) are removed. (**D**) cLTP stimulation induced pT286 but not pT305 or pT306 in WT cultured rat hippocampal neurons (*P* = 0.0480 for T286, *P* = 0.6522 for T305, and *P* = 0.7738 for T306; *n* = 3 wells). (**E**) cLTD stimulation induced phosphorylation of all three residues in WT rat neurons (unpaired two-tailed *t* test, *P* = 0.0168 for T286, *P* = 0.0078 for T305, and *P* = 0.0476 for T306; *n* = 4 wells).

Higher brain functions such as learning, memory, and cognition are thought to require long-term changes in synaptic strength such as hippocampal long-term potentiation (LTP) and depression (LTD), which are expressed largely through changes of synaptic AMPA-type glutamate receptors (AMPARs) (*1*–*5*). Induction of LTP and some forms of LTD requires Ca^2+^ influx through *N*-methyl-d-aspartate (NMDA)–type glutamate receptors (NMDARs), but with distinct stimulation patterns: Hippocampal LTP is typically induced by high-frequency stimulation (HFS) that causes brief but strong Ca^2+^ stimuli, whereas LTD is typically induced by low-frequency stimulation (LFS) that causes weak but prolonged Ca^2+^ stimuli (*6*–*8*). Ca^2+^-dependent activation of calcium/calmodulin-dependent protein kinase II (CaMKII) and its subsequent autophosphorylation at T286 (which generates Ca^2+^-independent “autonomous” kinase activity) (*9*–*12*) has long been associated with LTP (*13*–*15*) [for review, see (*16*–*19*)]. CaMKII pT286 appeared to be an ideal mechanism to mediate the LTP versus LTD decision, because like LTP, pT286 is rapidly induced by HFS in vitro (*20*, *21*), and frequency-dependent CaMKII activation has been observed also in live neurons (*22*, *23*). However, CaMKII pT286 is required not only for LTP but also for LTD (*24*) and thus cannot mediate the LTP versus LTD decision. Here, we show that this frequency detection by CaMKII is instead mediated via inhibitory autophosphorylation at T305/306. These residues are within the calmodulin (CaM) binding region of the regulatory domain (see Fig. 1A), and their phosphorylation is known to inhibit subsequent stimulation by Ca^2+^/CaM (*25*, *26*). Previous experiments with overexpression of CaMKII mutants indicated that pT305/306 could promote depression of synaptic strength (*27*–*29*); however, it has not been examined whether pT305/306 contributes to the physiologically induction of LTD or whether it even occurs in response to LTD stimuli. Our experiments here (i) elucidated the differential induction of pT305/306 in response to LTP- versus LTD-type stimulation in neurons and in vitro, (ii) showed the requirement for the frequency detection in the LTP versus LTD decision, (iii) determined the underlying autophosphorylation and inhibitory mechanisms within the 12meric CaMKII holoenzymes, and (iv) showed an additional requirement of pT305/306 in communicating the excitatory LTD stimuli also to inhibitory synapses. The CaMKIIα isoform is largely restricted to neurons, but the γ and δ isoforms are ubiquitously expressed and play important roles also outside the nervous system, including in regulating the cell cycle, immune response, and metabolism (*30*–*32*). Thus, our findings on the fundamentals of CaMKII holoenzyme regulation are highly relevant well beyond the specific functions in synaptic plasticity studied here.

## RESULTS

### CaMKII pT305/306 is selectively induced by LTD, but not LTP, stimuli

The major regulatory autophosphorylation sites within a CaMKII holoenzyme (*33*, *34*) are T286 and T305/306 (see Introduction and Fig. 1A). Increased pT286 was detected in hippocampal slices after electrical induction of LTP with HFS (2×, 1 s, 100 Hz) and after induction of LTD with LFS (15 min, 1 Hz) (Fig. 1B). By contrast, pT305/306 instead increased specifically only after stimulation of LTD but not LTP (Fig. 1B). Detection of biochemical changes after electrical stimulation was aided by using a monopolar stimulation electrode (which increases the stimulated area) on hippocampal CA1 mini-slices (which eliminates inclusion of nonstimulated other areas of the hippocampus; Fig. 1C). Similarly, using chemical stimulation of either LTP (cLTP; at 1 min after 100 μM glutamate, 10 μM glycine) or LTD [cLTD; at 5 min after 30 μM NMDA, 10 μM 6-cyano-7-nitroquinoxaline-2,3-dione (CNQX), 10 μM glycine] in hippocampal cultures induced a robust increase in pT286 (Fig. 1, D and E). Again, increased pT305/306 was detected selectively only after cLTD but not cLTP (Fig. 1, D and E). cLTD stimuli in slices also induced both pT286 and pT305/306 (fig. S1). Notably, pT286 was fully reversed at 5 min after cLTP stimuli in hippocampal cultures [consistent with previous findings; for review, see (*17*)], but after cLTD stimuli, pT286 remained elevated at this time point and pT305/306 was increased (fig. S2). In summary, pT286 is induced by both LTP and LTD stimuli, whereas pT305/306 is selectively induced by LTD stimuli in both cultured hippocampal neurons and in acute slices.

### CaMKII T305/306AV mutation impairs LFS-induced LTD but not HFS-induced LTP

**Fig. 2:**
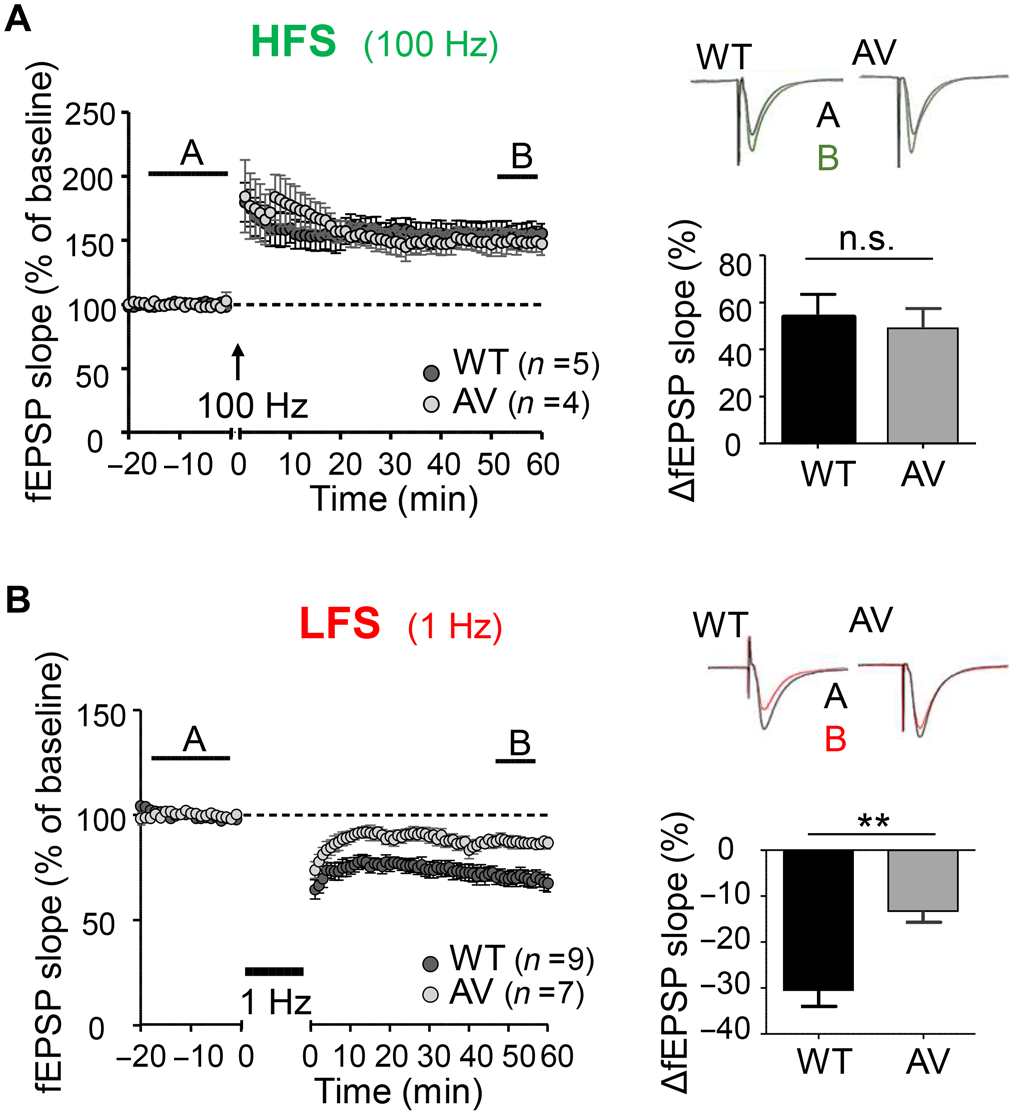
CaMKII pT305/306 is induced required for normal LTD but not LTP. Quantifications show means ± SEM. **P < 0.01. (**A**) LTP stimulation potentiates the CA3-CA1 Schaffer collateral pathway in both WT and T305/6AV CaMKII hippocampal slices (unpaired two-tailed *t* test, *P* = 0.6561, *n* = 5 and 4 slices). (**B**) Synaptic weakening induced by LTD stimulation is impaired in T305/6AV slices compared to WT (*P* = 0.0023, *n* = 9 and 7 slices).

CaMKII pT286 is required for both LTP and LTD at the excitatory hippocampal CA3 to CA1 synapse, as T286A mutation impairs both HFS-induced LTP (*15*) and LFS-induced LTD (*24*). To test the function of pT305/306, we used knock-in mice with the phospho-incompetent T305/306AV mutation (*35*). As expected, in electrophysiological field recordings of hippocampal slices, these mice showed normal HFS-induced LTP at the CA3 to CA1 synapse (Fig. 2A), consistent with previous results (*35*) and with the lack of pT305/306 in response to LTP stimuli (see Fig. 1, B and D). By contrast, LFS-induced LTD was significantly impaired by the T305/306AV mutation (Fig. 2B). Thus, at the excitatory model synapse tested here, pT286 is required for both LTP and LTD, whereas pT305/306 is instead selectively required only for normal expression of LTD. This indicates that pT305/306 but not pT286 mediates the LTP versus LTD decision by CaMKII.

### pT305/306 directs CaMKII movement during LTD from excitatory to inhibitory synapses

**Fig. 3:**
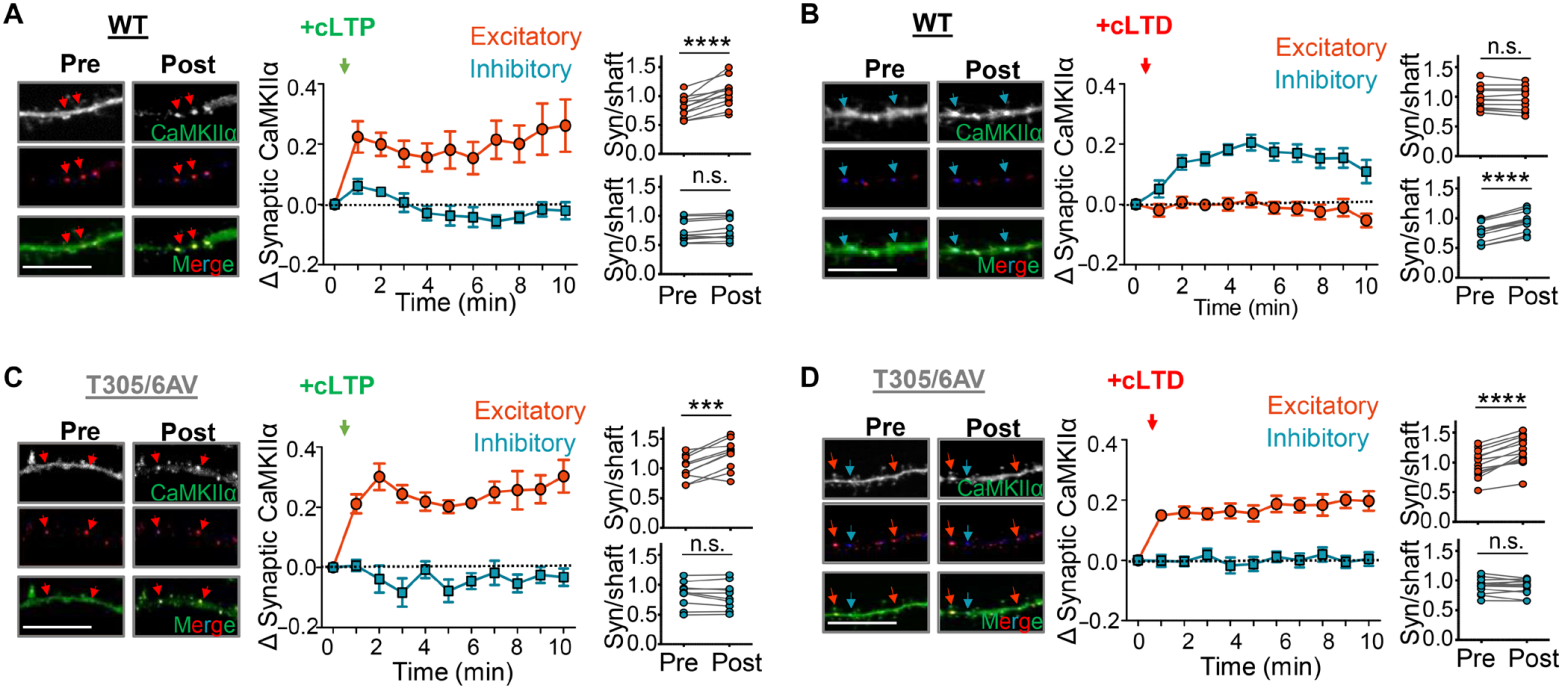
pT305/306 mediates LTD-induced CaMKII heterosynaptic targeting. Quantifications show means ± SEM. ***P < 0.001 and ****P < 0.0001. Imaging experiments followed movement of endogenous CaMKII (green) to excitatory synapses (PSD-95; red) or inhibitory synapses (gephyrin; blue) using intrabody labeling. Scale bars, 10 μm. (**A**) cLTP (100 μM glutamate/10 μM glycine, 1 min) induced CaMKII translocation to excitatory but not inhibitory synapses in cultured WT mouse hippocampal neurons (DIVs 14 to 17) (paired two-tailed *t* test, *P* < 0.0001 versus *P* = 0.0930, *n* = 14 neurons). (**B**) cLTD stimulation (30 μM NMDA/10 μM CNQX/10 μM glycine, 1 min) in WT neurons resulted in CaMKII movement to inhibitory but not excitatory synapses (*P* < 0.0001 versus *P* = 0.3543, *n* = 13 neurons). (**C**) Similar to WT, endogenous T305/6AV CaMKII targeted only excitatory synapses following cLTP (*P* = 0.009 versus *P* = 0.2228, *n* = 10 neurons). (**D**) In contrast to WT, CaMKII T305/6AV also targeted excitatory, and not inhibitory, synapses after cLTD (*P* < 0.0001 versus *P* = 0.6383, *n* = 13 neurons).

**Fig. 4:**
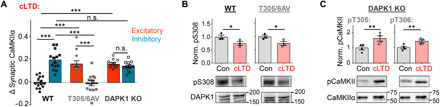
CaMKII pT305/306 and DAPK1 regulate LTD-related CaMKII targeting by independent mechanisms. Quantifications show means ± SEM. *P < 0.05, **P < 0.01, and ***P < 0.001. (**A**) Quantification of the change in synaptic CaMKII following cLTD stimulation (5 min after wash out) in WT, T305/6AV, and DAPK1 KO cultured hippocampal neurons. CaMKII in DAPK1 KO neurons targeted both excitatory and inhibitory synapses (one-way ANOVA, Tukey’s post hoc test; *n* = 14, 13, and 14 neurons). (**B**) cLTD-induced activation of DAPK1 via S308 dephosphorylation is not impaired in T305/6AV neurons (unpaired two-tailed *t* test, *P* = 0.0400 for WT and *P* = 0.0436 for AV, *n* = 3 wells per condition), as detected by immunoblot. (**C**) cLTD-induced autophosphorylation of CaMKII T305 and T306 is not impaired in DAPK1 KO neurons (unpaired two-tailed *t* test, *P* = 0.0291 for pT305 and *P* = 0.0205 for pT306, *n* = 4 wells per condition), as detected by immunoblot.

For monitoring movement of endogenous CaMKII after LTP versus LTD stimuli, we used our recently described method for simultaneous live imaging of CaMKII together with two marker proteins for excitatory versus inhibitory synapses, i.e., PSD-95 and gephyrin (*36*). This is enabled by simultaneous expression of three intrabodies that are labeled with three different fluorescent proteins. Basally, CaMKII was found throughout dendrites and dendritic spines (the sites of excitatory synapses), both in cultures from wild-type (WT) and T305/305AV mutant mice (Fig. 3, A to D). CaMKII WT accumulated further at excitatory synapses in response to cLTP stimuli and at inhibitory synapses after cLTD stimuli, as expected (Fig. 3, A and B). The endogenous CaMKII T305/306AV mutant showed the same movement as WT in response to cLTP stimuli (Fig. 3C), consistent with the normal LTP in these mice. However, after cLTD stimuli, the CaMKII T305/306AV mutant still moved to excitatory synapses and not to inhibitory synapses (Fig. 3C), i.e., it showed the opposite movement as WT after cLTD and instead a similar movement as seen after the opposing cLTP plasticity stimulus. Corresponding results were obtained also for overexpressed green fluorescent protein (GFP)–CaMKII WT versus T305/306AA mutant (fig. S3). Thus, pT305/306 is required during LTD both for suppressing CaMKII movement to excitatory synapses and for enabling CaMKII movement to inhibitory synapses (Fig. 4A).

### LTP specificity of CaMKII movement to excitatory synapses requires two independent LTD-specific suppression mechanisms

Our previous inhibitor studies indicated that activity of DAPK1 (death-associated protein kinase 1) is required for making the CaMKII movement to excitatory synapses LTP specific (*37*). Here, these findings were supported by experiments in cultures from DAPK1 knockout (KO) mice (Fig. 4A and fig. S4): Like the CaMKII T305/306AV mutation, DAPK1 KO prevented the suppression of CaMKII movement to excitatory synapses seen during cLTD in WT mice (Fig. 4A). However, in contrast to the CaMKII T305/306AV mutation, DAPK1 KO did not abolish the cLTD-induced CaMKII movement to inhibitory synapses (Fig. 4A).

**Fig. 5:**
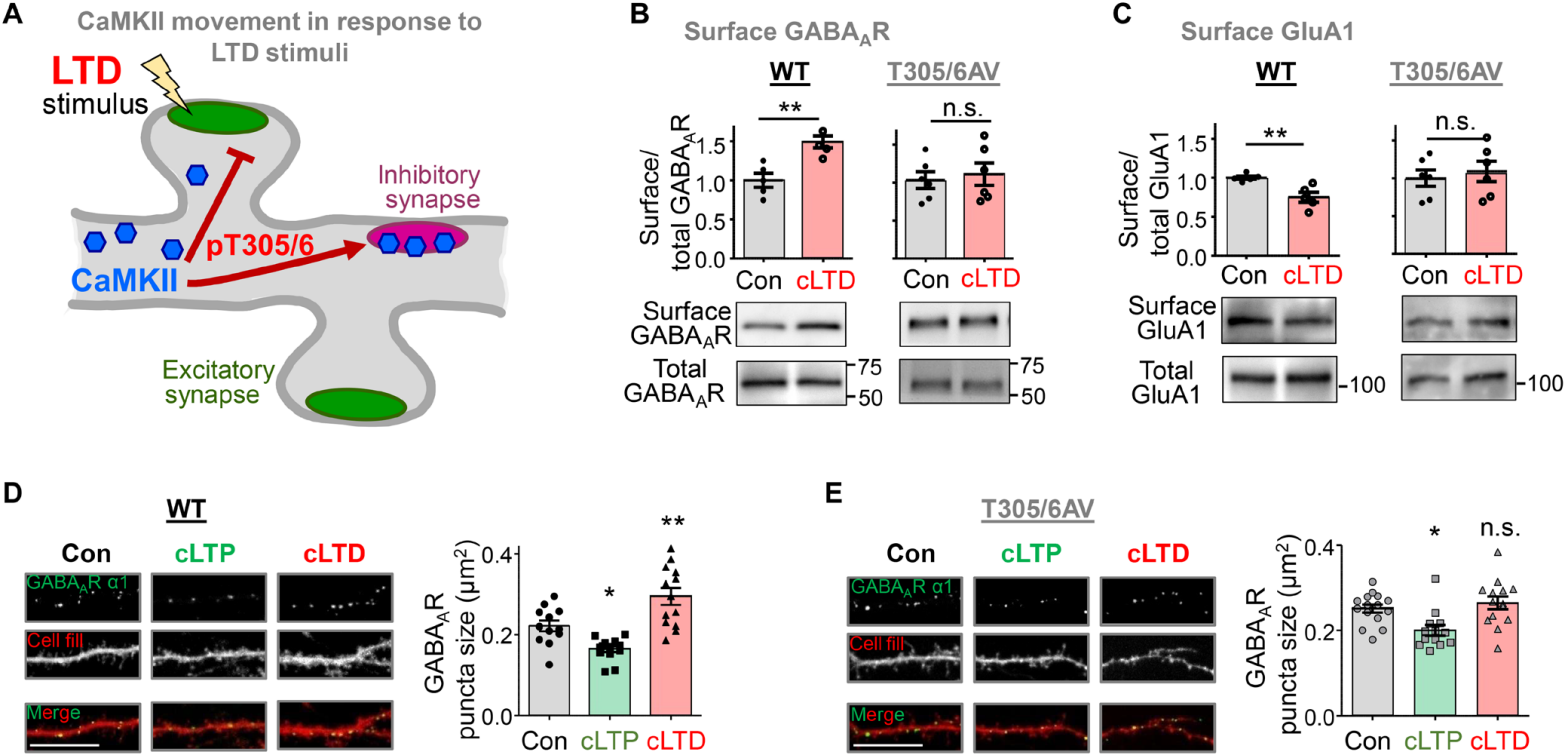
CaMKII pT305/306 promotes LTD-induced GABA_A_R insertion at inhibitory synapses. Quantifications show means ± SEM. *P < 0.05 and **P < 0.01. Scale bars, 10 μm. (**A**) Schematic of CaMKII movement in response excitatory LTD stimuli. The LTD-induced pT305/306 blocks CaMKII movement to glutamatergic excitatory synapses and instead enables movement to GABAergic inhibitory synapses. (**B**) cLTD stimulation increased surface GABA_A_R accumulation in WT, but not T305/6AV, CA1 mini-slices (unpaired two-tailed *t* test, *P* = 0.0033 for WT and *P* = 0.6760 for AV, *n* = 5 and 6 samples (two slices/sample), as detected by immunoblot after surface biotinylation. (**C**) cLTD stimulation decreased surface GluA1 accumulation in WT, but not T305/6AV, CA1 mini-slices (unpaired two-tailed *t* test, *P* = 0.0062 for WT and *P* = 0.6252 for AV, *n* = 5 and 6 samples (two slices/sample). (**D**) cLTP decreased surface GABA_A_R cluster size in nonpermeabilized neurons from WT mouse hippocampal cultures (fixed 5 min after washout), while cLTD increased the cluster size (fixed 20 min after washout) (one-way ANOVA, Tukey’s post hoc test versus control, *P* = 0.0273 for cLTP and *P* = 0.0046 for cLTD; *n* = 12, 12, and 13 neurons), as detected by immunocytochemistry. (**E**) By contrast, in nonpermeabilized neurons from T305/5AV hippocampal cultures, cLTD did not increase surface GABA_A_R clusters. However, cLTP still decreased surface GABA_A_R clusters (*P* = 0.0147 for cLTP and *P* = 0.7121 for cLTD; *n* = 15, 13, and 14 neurons).

The requirement for both DAPK1 and pT305/306 raised the possibility that both are steps along the same pathway. However, our results instead showed that the actions of DAPK1 and CaMKII pT305/306 are independent of each other: LTD induced DAPK1 activation (assessed by S308 dephosphorylation) also in T305/306AV mutant mice (Fig. 4B). and it induced CaMKII pT305/306 also in DAPK1 KO mice (Fig. 4C). Thus, suppression of CaMKII movement to excitatory synapses during LTD requires simultaneous engagement of two independent mechanisms; of these two mechanisms, only pT305/306 is additionally required to enable CaMKII movement to inhibitory synapses (as illustrated in Fig. 5A).

### pT305/306 promotes iLTP at inhibitory synapses in response to excitatory LTD

While the CaMKII movement to excitatory synapses during LTP is input specific (as it occurs specifically to the stimulated synapse) (*38*), the LTD-induced CaMKII movement to inhibitory synapses is by definition heterosynaptic (as it is induced by stimulation of a different synapse type, excitatory synapses; Fig. 5A). Functionally, excitatory LTD stimuli can cause induction of LTP at inhibitory synapses (iLTP) (*39*, *40*). Our results indicate that this heterosynaptically induced iLTP is mediated by CaMKII pT305/306: cLTD stimuli induced an increase in the surface expression of the inhibitory γ-aminobutyric acid type A (GABA_A_) receptors, but only in hippocampal slices from WT mice, and not from T305/306AV mice (Fig. 5B). At excitatory synapses, the cLTD stimuli induced a decrease in surface expression of the AMPAR subunit GluA1, but again only in slices from WT mice and not from T305/306AV mice (Fig. 5C), as expected based on the impairment of excitatory LTD in these mutant mice. The experiments in slices were done by receptor surface biotinylation. In addition, we labeled surface GABA_A_ receptors (GABA_A_ Rs) in hippocampal cultures by immunocytochemistry without cell permeabilization (to hide the epitopes of intracellular receptors; Fig. 5, D and E, and fig. S5). Again, cLTD stimuli induced an increase in GABA_A_ R surface expression in WT cultures (Fig. 5D) but not in T305/306AV cultures (Fig. 5E). By contrast, cLTP stimuli caused a reduction of GABA_A_ R surface expression in both WT and mutant cultures (Fig. 5, D and E), again consistent with the normal LTP in the T305/306AV mice. Notably, the effects on GABA_A_ R surface expression after both cLTP and cLTD manifested in corresponding changes in both number and size of the puncta detected by the surface stain; however, the cLTD-specific effect of the T305/306AV mutant manifested only in the size (Fig. 5, D and E) but not in the number of the puncta (fig. S5, A and B), suggesting that pT305/306 regulates only existing inhibitory synapses.

### pT305/306 curbs activity of pT286 CaMKII also in the absence of Ca^2+^

**Fig. 6:**
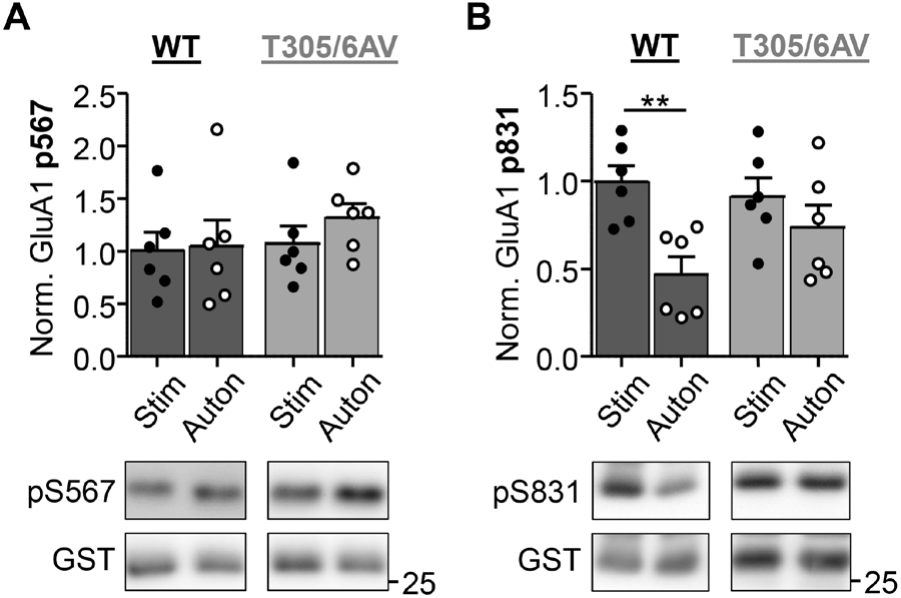
Effect of pT305/306 on GluA1 phosphorylation at S567 versus S831 after defined biochemical stimulation in the test tube. Quantifications show means ± SEM. **P < 0.01. (**A**) Phosphorylation of the LTD-related GluA1 S567 site by pT286-CaMKII (10 nM kinase subunits) with Ca^2+^/CaM present [stimulated (Stim)] or absent [autonomous (Auton)]. No differences in pS567 were detected between autonomous versus stimulated targeting by either WT or T305/306AV (two-way ANOVA, Bonferroni post hoc test, *P* = 0.5249 for WT and *P* = 0.0730 for T305/306AV, *n* = 5 reactions), although the mutant showed a trend toward increased pS567 with autonomous activity. (**B**) Phosphorylation of the LTP-related GluA1 S831 site by pT286-CaMKII (10 nM kinase subunits) with Ca^2+^/CaM present (stimulated) or absent (autonomous). Enhanced pS831 under stimulated versus autonomous conditions was seen with CaMKII WT (two-way ANOVA, Bonferroni post hoc test, *P* = 0.0039, *n* = 5) but not with the T305/306AV mutant (*P* = 0.7519, *n* = 5 reactions).

At excitatory synapses, CaMKII mediates phosphorylation of GluA1 at two distinct sites: pS831 promotes the LTP-related increase in single channel conductance (*41*, *42*); pS567 is induced during LTD and instead promotes a decrease in synaptic channel number (*24*, *43*). This stimulus-dependent substrate-site selection on GluA1 results from S567 being an unusual substrate type: Phosphorylation by pT286 CaMKII was significantly further enhanced by additional Ca^2+^/CaM stimulation in case of S831 and other regular substrates, but such enhancement of stimulated over autonomous activity was not seen in case of S567 or other LTD-related substrates, resulting in equal or higher phosphorylation by autonomous activity in the absence of Ca^2+^ (*12*, *24*, *44*). As removal of Ca^2+^ from autonomous CaMKII quickly induces pT305/306 (see also fig. S6), we decided to test whether pT305/306 is required for the elevated level of autonomous phosphorylation of S567. However, T305/306AV mutation did not reduce the autonomous phosphorylation of S567 in biochemical assays in vitro; if any, it appeared slightly enhanced (Fig. 6A). Instead, for S831 phosphorylation, T305/306AV mutation abolished the Ca^2+^/CaM-induced enhancement of phosphorylation by pT286 CaMKII (Fig. 6B), indicating that pT305/306 is the reason why pT286 CaMKII is not fully active in the absence of Ca^2+^. Thus, pT305/306 not only prevents further stimulation of the autonomous activity of pT286 CaMKII but also directly contributes to the lower activity of autonomous pT286 CaMKII compared to fully Ca^2+^/CaM-stimulated CaMKII.

### pT286 versus pT305/306 in response to biochemical CaMKII stimulation in the test tube

**Fig. 7:**
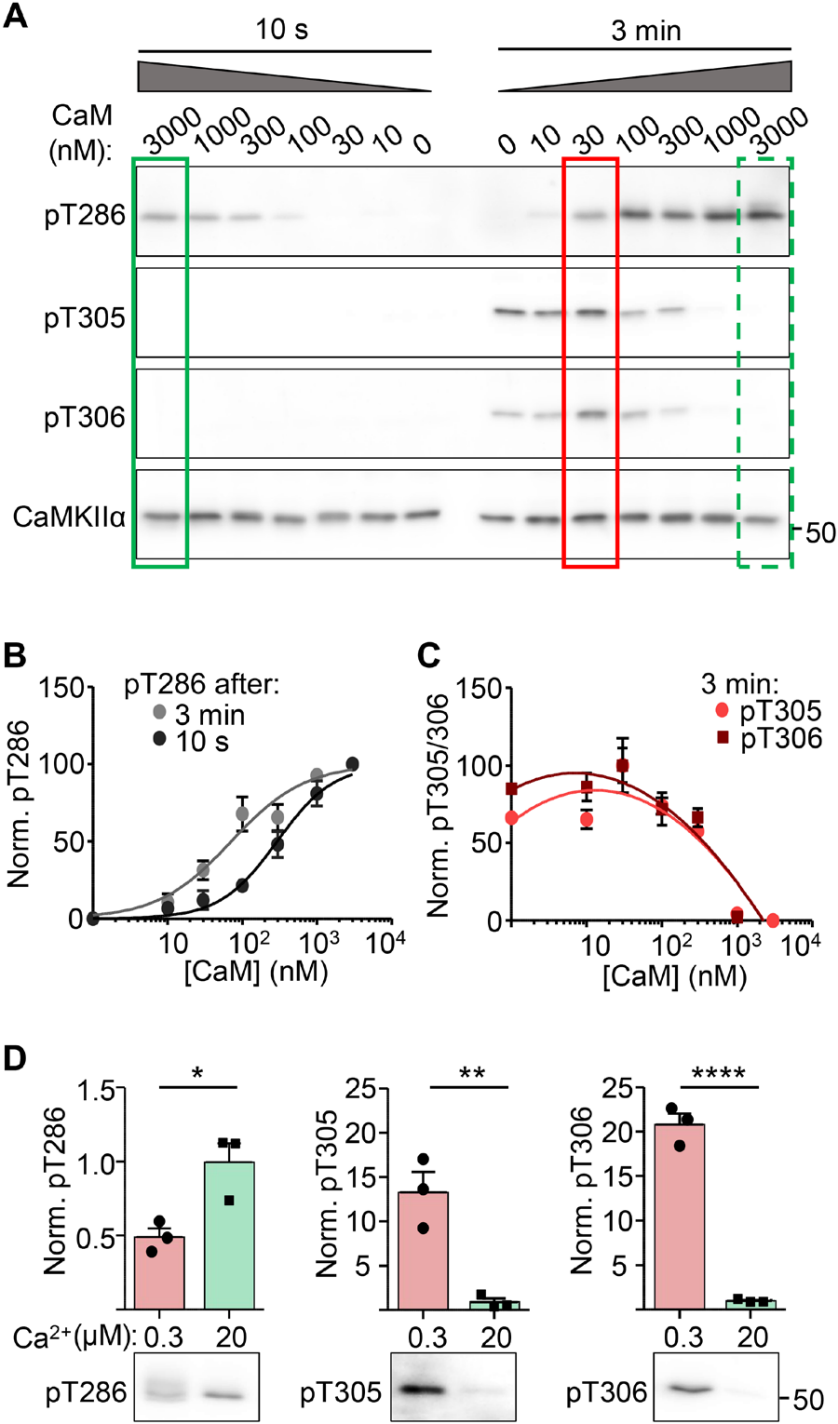
LTD- and LTP-related CaMKII phosphorylation induced by defined biochemical stimulation in the test tube. Quantifications show means ± SEM. *P < 0.05, **P < 0.01, and ****P < 0.0001. (**A**) Representative immunoblots of CaMKII (100 nM kinase subunits) after brief (10 s) or prolonged (3 min) stimulation with 2 mM Ca^2+^ and varied CaM (0 to 3 μM CaM). High CaM concentrations favored pT286 and suppressed pT305/306 (green boxes, LTP-related), with the latter instead favored by prolonged stimulation with low CaM (red box; LTD-related). (**B**) Quantification of increased pT286 with increasing Ca^2+^/CaM. Prolonged stimuli induced a leftward shift in the concentration-dependent response. (**C**) pT305/306 increased from 0 to 0.03 μM CaM, then decreased with higher CaM concentration. pT305/306 was detected after prolonged (3 min) but not brief (10 s) stimulation. (**D**) Representative immunoblots and quantification of CaMKII (10 nM kinase subunits) after brief, strong stimuli (10 s, 20 μM Ca^2+^, 1 μM CaM) versus weak, prolonged stimuli (3 min, 0.3 μM Ca^2+^, 1 μM CaM). pT286 showed a greater increase after high Ca^2+^ compared to low Ca^2+^ stimuli (unpaired two-tailed *t* test; *P* = 0.0235, *n* = 3). Increased pT305/306 was only detected after low Ca^2+^ stimuli (unpaired two-tailed *t* test, *P* = 0.0057 for pT305 and *P* < 0.0001 for pT306, *n* = 3 reactions).

After establishing a role of pT305/306 in the LTP versus LTD decision, we wanted to elucidate the underlying biochemical mechanisms. Thus, we determined the effect of different biochemical stimulation types on purified CaMKII in vitro. Similar to the neuronal response to LTP versus LTD stimuli (see Fig. 1), pT286 in vitro was promoted both by stimuli mimicking LTP (brief high Ca^2+^/CaM) or LTD (prolonged low Ca^2+^/CaM), while pT305/306 instead specifically required stimuli mimicking LTD (Fig. 7A). For pT286, the LTD-related prolonged reaction times lowered the requirement for Ca^2+^/CaM (Fig. 7B); the corresponding apparent reduction of the half maximal effective concentration (EC_50_) was expected simply based on more pT286 during the longer reaction times. For T305/306, minimal or no phosphorylation was seen after any brief stimulation (10 s); at prolonged times (3 min), it was stimulated by low Ca^2+^/CaM (0.03 to 0.01 μM) but completely suppressed by the LTP-related high Ca^2+^/CaM (1 to 3 μM) (Fig. 7C). The suppression of pT305/306 by high Ca^2+^/CaM is consistent with the overlapping sites on the regulatory domain, making CaM binding and pT305/306 mutually exclusive (see Fig. 1A). The stimulation of pT305/306 by low Ca^2+^/CaM is consistent with the effect of previous pT286, which markedly accelerates pT305/306 compared to basal conditions (fig. S6). In these experiments, stimulation strength was adjusted by the concentration of CaM. However, the same results were also obtained when LTP versus LTD stimuli were instead mimicked by varying Ca^2+^ concentrations, specifically 20 μM versus 0.3 μM Ca^2+^ at 1 μM CaM [Fig. 7D and fig. S7A; conditions that more closely mimic the regulation in neurons (*8*)]. Together, these results suggest that the CaMKII holoenzyme is sufficient to distinguish between LTP and LTD stimuli, i.e., without the requirement of cellular components other than Ca^2+^ and CaM.

### Fast pT305/306 within CaMKII holoenzymes can occur trans-subunit

**Fig. 8:**
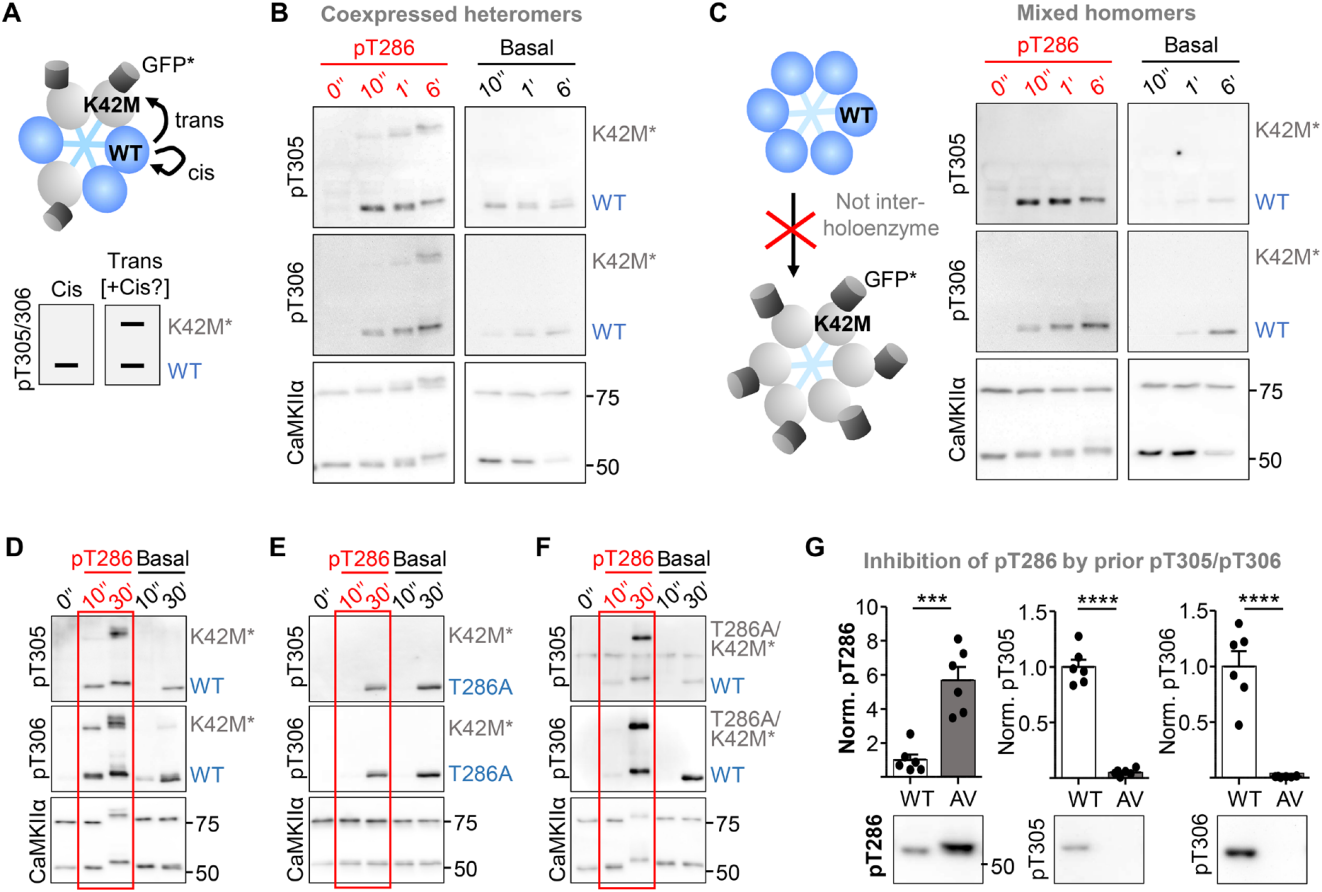
Autonomous CaMKII targets pT305/306 through trans-subunit phosphorylation. Cis- versus trans-subunit T305/306 phosphorylation within CaMKII holoenzymes was assessed in biochemical assays without Ca^2+^, either with prior pT286 (red) or without. (**A**) Schematic of the assay: K42M blocks kinase activity and GFP tag allows distinction on immunoblots. (B) With prior pT286 stimulation, pT305/306 occurred in trans. Slow basal pT305/306 occurred in cis only. (**C**) pT305/306 occurred within but not between holoenzymes, as homomeric WT holoenzymes did not phosphorylate homomeric K42M-GFP holoenzymes. Homomeric holoenzymes were created by separate expression, with subsequent mixing of extracts. Heteromeric holoenzymes in all other panels were created by coexpressing CaMKII forms within the same cells. (**D** to **F**) The requirements for trans phosphorylation tested with different mutant combinations (in addition to GFP-tagged K42M). (D) The pT305/306 trans phosphorylation was confirmed and (E) required prior pT286, as it was abolished by T286A mutation on the kinase subunit. However, (F) pT305/306 trans phosphorylation did not require pT286 on the substrate subunit, as it still occurred after T286A mutation on the GFP-tagged K42M mutant. (**G**) Previous basal pT305/306 prevented subsequent Ca^2+^/CaM-stimulated pT286 in WT but not T305/6AV. As expected, basal prereaction generated pT305/306 only in WT but not T305/306AV (***P = 0.0002 and ****P < 0.0001; unpaired two-tailed *t* test; *n* = 5 reactions). Loading control shown in fig. S7B.

Next, we decided to elucidate the holoenzyme mechanism by which pT305/306 occurs. For this purpose, we used pairs of CaMKII mutants in vitro, after coexpression in human embryonic kidney (HEK) cells to allow integration into the same holoenzymes (Fig. 8A). The kinase dead K42M mutant is always an obligate substrate subunit (as it cannot phosphorylate itself or other subunits in the holoenzyme). In addition, the K42M mutant was labeled with a GFP tag, to allow for distinction during phospho-detection by immunoblot (Fig. 8A): Any phosphorylation of the tagged K42M subunit must have occurred in trans (i.e., between subunits) as it could not have occurred in cis (i.e., within the same single subunit). At the CaMKII concentrations used, all autophosphorylation at T305/306 occurred within individual holoenzymes and not between them (Fig. 8, B and C). Within holoenzymes, the slow basal phosphorylation (reaction rate of ~1 per 20 min) occurred only in cis (Fig. 8B) and appeared to be more prevalent for T306 compared to T305 (fig. S6), consistent with previous reports (*45*). By contrast, the faster phosphorylation that is induced by dissociation of Ca^2+^/CaM from pT286 CaMKII has not been examined previously and was found here to occur also in trans (Fig. 8, B and D). This trans phosphorylation required pT286 on the kinase subunit, as it was abolished by a T286A mutation on the un-tagged active subunit (Fig. 8E). As expected, the same trans phosphorylation was also detected when the GFP tag was switched from the kinase dead K42M substrate subunit to the active kinase subunit (fig. S6A) and when the GFP tag was completely eliminated (fig. S6B; in a reaction with a pair of T286D T305/306AV mutant kinase subunit and K42M mutant substrate subunit, in which any pT305/306 can only occur in trans). These experiments demonstrated a trans mechanism but did not rule out an additional cis mechanism. An additional cis mechanism also for the fast phosphorylation by pT286 autonomous kinase was expected, as this was the exclusive mechanism for the slow phosphorylation by basal kinase activity (see above). An additional cis-subunit phosphorylation was detected using monomeric CaMKII (fig. S6C); this was done after initial pT286 reaction at high concentrations (to enable pT286 in the monomer) (*46*), followed by a pT305/306 reaction after dilution (to a level that allows cis phosphorylation but suppressed trans holoenzyme reactions, as shown in Fig. 8C). Together, whereas the slow basal pT305/306 occurs only cis-subunit, the pT286-dependent fast pT305/306 can occur by both cis- and trans-subunit autophosphorylation.

### The pT305/306 trans-subunit mechanism allows suppression of pT286 in a holoenzyme

As fast phosphorylation of T305/306 requires previous phosphorylation of T286 (followed by dissociation of Ca^2+^/CaM), pT305/306 was thought to occur essentially exclusively on a triple-phosphorylated pT286/305/306 CaMKII, which would then still show Ca^2+^-independent autonomous activity (*16*). By contrast, the pT305/306 trans-subunit mechanism could allow completely shutting down activity of a neighboring subunit; that is unless the trans mechanism does additionally require pT286 also on the substrate subunit. However, pT286 was required only on the active subunit (Fig. 8E) but not on the tagged kinase dead K42M obligate substrate subunit (Fig. 8F). If any, trans phosphorylation appeared to be favored on neighboring subunits without pT286, as the trans phosphorylation was diminished for a phospho-mimetic T286D mutation (fig. S6D). Thus, while the known pT305/306 cis phosphorylation of a pT286 subunit reduces the level of autonomous activity, the trans phosphorylation described here can completely shut down activity of a neighboring non-pT286 subunit. In addition, such trans phosphorylation should also prevent future pT286 on this subunit, because pT286 requires CaM binding also to the phosphorylated subunit. pT305/306 phosphorylation caused the expected feed-forward inhibition of subsequent pT286 (Fig. 8G and fig. S7B), thereby curbing pT286 to a submaximal level (as observed after LTD stimuli; see Fig. 1).

**Fig. 9:**
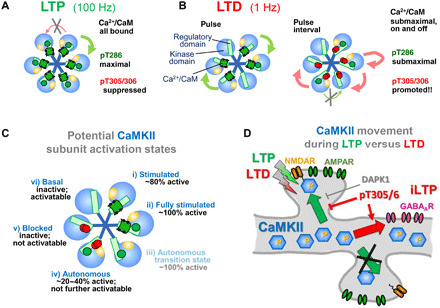
Schematic of CaMKII regulation. 12meric holoenzymes depicted as hexamers. (**A**) LTP stimuli induce maximal pT286. At 100 Hz, pulse intervals are shorter than the Ca^2+^/CaM dissociation time, promoting complete Ca^2+^/CaM binding to all subunits. (**B**) LTD stimuli induce submaximal pT286 during the 1-Hz pulses. CaM dissociation during the pulse intervals triggers pT305/306 in cis (limiting the autonomous activity on the pT286 subunit) and in trans (completely blocking activity and subsequent pT286 of this subunit). Low amplitude cLTD stimuli cause similar effects, due to submaximal on-and-off equilibrium Ca^2+^/CaM binding. (**C**) Cross-regulation of CaMKII activity by Ca^2+^/CaM, pT286, and pT305/306. (i) Ca^2+^/CaM stimulates kinase activity but (ii) additional pT286 causes full stimulation (*55*). (iii) The pT286 subunit remains active (autonomous) after dissociation of CaM, but this quickly triggers (iv) cis pT305/306, reducing the level of autonomous activity. (v) trans pT305/306 prevents future activation of that subunit. (vi) In the basal state (naïve or after dephosphorylation), subunits are inactive but competent for stimulation. (**D**) LTP and LTD stimuli both induce pT286 (albeit different levels). During LTP, CaMKII translocates to excitatory synapses in an input specific manner; this movement is prevented during LTD by pT305/306 and DAPK1. In addition, LTD-induced pT305/306 causes CaMKII movement to inhibitory synapses, which mediates inhibitory iLTP after excitatory LTD stimuli.

Together, the complex cross-regulation of pT286 and pT305/306 identified here (and illustrated schematically in Fig. 9 and fig. S9) provides the holoenzyme mechanisms for computation of input stimuli, which enables the differential frequency response in the LTP versus LTD decision.

## DISCUSSION

This study revealed previously unknown fundamentals of CaMKII regulation by inhibitory autophosphorylation at T305/306. In addition, we show that these regulatory principles are required for (i) the homosynaptic signal computation that leads to the LTP versus LTD decisions at excitatory synapses and (ii) the heterosynaptic communication of these decisions also to inhibitory synapses. Thus, the inhibitory CaMKII regulation mechanisms are essential for the complex synaptic plasticity signaling that is required for higher brain functions. However, the mechanisms also provide important precedent for understanding CaMKII signaling in general, including outside of the nervous system.

It is well established that each subunit within the 12meric CaMKII holoenzyme is activated separately by direct binding of Ca^2+^/CaM [for review, see (*16*)]. However, when Ca^2+^/CaM binds to two neighboring subunits, it additionally induces a fast trans-subunit autophosphorylation at T286 (*46*, *47*), which generates autonomous kinase activity that outlasts the initial Ca^2+^ stimulus. Then, dissociation of Ca^2+^/CaM exposes T305/306 for phosphorylation by the autonomous CaMKII. Our results revealed that this pT305/306 has unexpected mechanisms and consequences. In contrast to pT286, the pT305/306 reaction can occur by both cis- and trans-subunit mechanisms. Although the occurrence of the cis-mechanism has been recognized previously, its major consequence has not: In addition to inhibiting subsequent Ca^2+^/CaM binding, pT305/306 directly contributes to curbing the level of the Ca^2+^-independent autonomous activity to a level that is lower than the maximal Ca^2+^/CaM-stimulated activity. Thus, it is the cis-pT305/306 reaction that distinguishes the lower level of prolonged autonomous CaMKII activity from the maximal CaMKII activity during acute Ca^2+^ stimuli. These distinct levels of activity have been recognized before but were misinterpreted as an entirely intrinsic property of pT286-induced autonomy (*12*, *24*). By contrast, our results indicate that pT286 and Ca^2+^/CaM may not substantially differ in the degree of opening the inhibitory gate that is provided by the regulatory domain. Instead, pT305/306 actively contributes to some level of direct inhibition, independently from preventing Ca^2+^/CaM binding. However, inhibition of Ca^2+^/CaM binding remains an important regulatory feature of pT305/306, specifically through its previously unrecognized trans-subunit mechanism: This trans-subunit reaction can induce pT305/306 on neighboring subunits that are not autonomous, thereby completely preventing their activation. The trans-pT305/306 reaction requires previous pT286 on the neighboring subunit that acts as the kinase but then leads also to a feed-forward inhibition of subsequent pT286 on these pT305/306-only subunits. Together, these mechanisms enable a wide array of finely tuned possible states for each CaMKII holoenzyme, with each state tightly linked to the inducing Ca^2+^ stimulus. For instance, for a trans-pT305/306 to completely shut down a kinase subunit, the Ca^2+^ stimulus must be sufficiently high to induce some pT286 within the holoenzyme (which requires Ca^2+^/CaM binding to two neighboring subunits) but sufficiently low to not induce pT286 on all of the neighboring subunits. Such finely graded responses may be of particular importance for the complex neuronal signaling processes that underlie cognitive brain functions. However, as we were able to generate similarly graded responses also in a highly reductionist biochemical system in vitro, it may be functionally important also in other in vivo systems that may require less complex signal computation.

Our results show that the frequency detection in the LTP versus LTD decision requires the interplay between the pT286 and pT305/306 mechanisms within the CaMKII holoenzyme. Like LTP, pT286 is favored by HFS (*20*). However, pT286 can also be induced by LFS, at least when it is sufficiently prolonged (*20*). These effects are based on the requirement of two CaM molecules for the reaction [for review, see (*16*)]: Higher frequencies (with spike intervals shorter than the CaM dissociation time) increase the likelihood of two CaM molecules binding to two neighboring subunits and thereby the likelihood of pT286. However, at lower frequencies, the pT286 likelihood is not zero and increasing the spike number can still lead to maximal pT286. LTD stimuli are typically prolonged, such as 15-min stimulation at 1 Hz, which delivers 900 pulses; this is in contrast to the 100 pulses delivered when LTP is induced by a 1-s stimulus at 100 Hz. Thus, despite its preferential induction by higher frequencies, pT286 by itself cannot distinguish between the short high-frequency stimuli that lead to LTP and the prolonged low-frequency stimuli that lead to LTD. Instead, distinguishing LTP from LTD stimuli is enabled by additional pT305/306: During HFS, pT305/306 is suppressed by the continuously bound CaM, whereas LFS allows the intermittent dissociation of CaM that triggers pT305/306 by the autonomous CaMKII. Notably, the same mechanism can also distinguish between stimulation amplitudes, with pT305/306 effectively suppressed by high but not low Ca^2+^/CaM concentrations. This frequency/amplitude equivalency in CaMKII regulation may be important for continuity of postsynaptic CaMKII signaling, as the frequency of presynaptic stimuli is likely to be modulated into a postsynaptic amplitude response (at least for higher stimulation frequencies that significantly exceed 10 Hz). In addition, this CaMKII equivalency may help explain why LTD can be induced equivalently by low frequencies (in electrically induced LTD) and by low amplitudes (in chemically induced LTD) (*7*, *48*).

Generation of pT305/306 was favored by electrical LTD stimuli in hippocampal slices (with low frequency), by cLTD stimuli in neuronal cultures [with low-level NMDA receptor (NMDAR) activation], and by mimicking the mild LTD stimuli in the test tube (by prolonged stimulation at low Ca^2+^/CaM concentrations). The biochemical mechanisms described above enable both this stimulation preference and the resulting curbed CaMKII activity, including curbed pT286. Notably, pT286 is required for both LTP and LTD (*15*, *24*); however, LTD appears to require a limitation on the resulting CaMKII activity, which is provided by pT305/306. This enables the previously described preferential phosphorylation of LTD-related substrates by autonomous CaMKII in the absence of Ca^2+^/CaM (*24*, *44*). In addition to these intrinsic mechanistic differences between LTP- and LTD-related substrates, substrate selection is likely guided by subcellular targeting. Normal LTP requires CaMKII movement to excitatory synapses, mediated by regulated binding to the NMDAR subunit GluN2B (*49*, *50*). By contrast, LTD requires active suppression of this CaMKII movement (*37*). We showed here that this suppression requires both DAPK1 (likely by competitive binding to GluN2B) (*37*) and CaMKII pT305/306 (which has been shown to reduce GluN2B binding in vitro) (*28*): Elimination of either one of the two independent mechanisms abolished the suppression. Thus, either one of the two mechanisms alone is not sufficient to block CaMKII movement, indicating that CaMKII has a strong intrinsic propensity to move to excitatory synapses even during the weaker LTD stimuli.

Notably, the CaMKII T305/306AV mutation significantly reduced excitatory LTD in hippocampal slices but, in contrast to acute CaMKII inhibition (*24*), did not abolish LTD completely. A similar apparent discrepancy has been described for LTP, which is also completely blocked by acute CaMKII inhibition (*13*, *51*) but only reduced by genetic CaMKIIα KO (*35*, *52*) or by genetic prevention of CaMKII binding to GluN2B (*50*). At least for LTP, this could potentially be explained by additional contributions of the related CaMKIIβ isoform (*53*), which would also be targeted by the inhibitors. However, both LTP and LTD are completely blocked by T286A mutation on the CaMKIIα isoform alone (*15*, *24*), suggesting an additional possibility: Activity of the CaMKIIα isoform may be necessary and sufficient for LTD, but the different genetic CaMKII mutations may show different levels of compensatory effects (which is commonly recognized as a potential complication with transgenic animals).

LTD stimuli cause not only active suppression of CaMKII movement to excitatory synapses but also cause an LTD-specific CaMKII movement to inhibitory synapses (*36*, *54*). This movement also required pT305/306 (but not DAPK1), explaining the specific occurrence only during LTD but not LTP. The relevant binding partner at inhibitory synapses is currently not known, but the requirement of pT305/306 may provide an approach for screening for relevant binding partners at inhibitory synapses in future studies. CaMKII has been shown to mediate iLTP at inhibitory synapses in response to excitatory LTD stimuli (*40*, *54*). Our results showed that such LTD-induced increase in GABA_A_R surface expression requires pT305/306, indicating that iLTP requires the physical movement of CaMKII to inhibitory synapses, similar as LTP at excitatory synapses. However, whereas CaMKII accumulation during LTP occurs at the stimulated excitatory synapse in an input-specific manner (*38*), the LTD-induced accumulation at inhibitory synapses instead represents a form of heterosynaptic communication. This heterosynaptic communication can then coordinate the decrease in synaptic strength at excitatory synapses with an increase at inhibitory synapses to result in an even greater shift the excitation/inhibition balance.

Together, our results show how CaMKII pT305/306 determines the direction of homosynaptic plasticity at excitatory synapses and its heterosynaptic communication to inhibitory synapses. These are important mechanisms in neuronal computations that ultimately underlie higher brain functions such as learning, memory, and cognition. Equally important, however, may be the elucidation of fundamental regulatory principles within the CaMKII holoenzyme that likely govern CaMKII-dependent functions also beyond the nervous system.

## MATERIALS AND METHODS

### Material and DNA constructs

The following antibodies were used: GABA_A_R α1 (1:1000; Synaptic Systems, 224 211), GluA1 (Millipore, AB1504), GluA1 pS845 (1:1000; PhosphoSolutions, p1160-845), GluA1 pS831 (1:1000, PhosphoSolutions, p1160-831), CaMKII pT286 (1:1000, PhosphoSolutions, p1005-286), CaMKII pT305 (1:800; Assay BioTech, A0005; but see fig. S2 and the note below), CaMKII pT306 (1:800; PhosphoSolutions, p1005-306), DAPK1 (1:1000; Sigma-Aldrich, D1319), DAPK1 pS308 (1:1000, Sigma-Aldrich, D4941), GluA1 pS567 (1:1000, provided by K. Roche) (*43*), and CaMKIIα (1:5000; CBα2, made in-house), 2° anti-rabbit (1:600; GE Healthcare, NA934V), anti-mouse secondary (1:10,000, GE Healthcare, NA931V), and Alexa Fluor 647 anti-mouse (1:5000; Molecular Probes, A21236). For immunoblots, antibody incubations contained 5% milk, with the exception of incubations with DAPK1 and DAPK1 p308 antibodies, which contained 5% bovine serum albumin (BSA) instead. For immunocytochemistry, GABA_A_R α1 and Alexa Fluor 647 anti-mouse were both diluted in 5% BSA/phosphate-buffered saline (PBS).

Note that all CaMKII pT305 antibody used was purchased before April 2020, unless indicated otherwise (i.e., in fig. S2). We and others have successfully used this antibody previously (*28*, *55*, *56*). However, two different samples of the same antibody that were obtained after August 2020 (for additional experiments during the revision) failed to successfully detect pT305 after cLTD stimuli in neurons, even, at fourfold, the antibody concentrations typically used and although pT306 was successfully detected in the same samples (fig. S2). A faint signal was obtained only for purified CaMKII that was phosphorylated in vitro (fig. S2).

The expression vectors for the GFP-labeled FingR intrabodies targeting CaMKIIα, PSD-95, and gephyrin were provided by D. Arnold (University of Southern California, Los Angeles, CA, USA) as previously characterized (*57*, *58*). As we have described recently (*36*), the fluorophore label was exchanged using Gibson Assembly to contain the following tags in place of GFP: CaMKIIα-FingR-YFP2, PSD-95–FingR-mCh, and gephyrin-FingR-mTurquois. Mutated CaMKII constructs were created with complementary mutagenic nucleotides for polymerase chain reaction (PCR) amplification of the WT CaMKII (GFP-tagged or GFP-untagged) plasmid using PfuPolymerase (Agilent) and subsequent digestion with DpnI to remove nonmutated, methylated template DNA before transformation of the PCR product into competent *Escherichia coli* (made in-house).

### Vertebrate animal models

All animal procedures were approved by the University of Colorado Institutional Animal Care and Use Committee and carried out in accordance with National Institutes of Health (NIH) best practices for animal use. All animals were housed in ventilated cages on a 12-hour light/12-hour dark cycle and were provided ad libitum access to food and water. Mixed sex WT or mutant mouse littermates (on a C57BL/6 background) from heterozygous breeder pairs were used for slice electrophysiology and biochemistry. Mixed sex pups from homozygous mice [postnatal day 1 (P1) to P2] or Sprague-Dawley rats (P0) were used to prepare dissociated hippocampal cultures for imaging and biochemistry. T305/306AV and DAPK1 KO mice are described previously (*35*, *59*). The T305/306AV mice were provided by Y. Elgersma (Erasmus MC, Rotterdam); the DAPK1 KO mice were provided by T. H. Lee (Harvard University) with permission by A. Kimchi (Weizman Institute for Science).

### Hippocampal slice preparation from mouse

WT and mutant mouse hippocampal slices were prepared using P13 to P17 mice, an age at which NMDAR-dependent LTD is robust (*60*). Isoflurane anesthetized mice were rapidly decapitated, and the brain was dissected in ice-cold high sucrose solution containing 220 mM sucrose, 12 mM MgSO_4_, 10 mM glucose, 0.2 mM CaCl_2_, 0.5 mM KCl, 0.65 mM NaH_2_PO_4_, 13 mM NaHCO_3_, and 1.8 mM ascorbate. Transverse hippocampal slices (400 μm) were made using a tissue chopper (McIlwain) and transferred into 32°C artificial cerebral spinal fluid (ACSF) containing 124 mM NaCl, 2 mM KCl, 1.3 mM NaH_2_ PO4, 26 mM NaHCO_3_, 10 mM glucose, 2 mM CaCl_2_, 1 mM MgSO_4_, and 1.8 mM ascorbate. All solutions were recovered in 95% O_2_/5% CO_2_ for at least 1.5 hours before experimentation. Preparation of hippocampal CA1 mini-slices was performed as described above with additional cuts to isolate the CA1 region (see Fig. 1C).

### Hippocampal culture preparation from mouse or rat

To prepare primary hippocampal neurons from WT or mutant mice, hippocampi were dissected from mixed sex mouse pups (P1 to P2), dissociated in papain for 30 min, and plated at 200 to 300,000 cells/ml for imaging or 500,000 cells/ml for biochemistry. To prepare rat neurons, hippocampi were dissected from mixed set rat pups (P0), dissociated in papain for 1 hour, and plated at 100,000 cells/ml for imaging. At days in vitro (DIVs) 12 to 14, neurons were transfected with 1 μg of total cDNA per well using Lipofectamine 2000 (Invitrogen) and then imaged or treated and fixed 2 to 3 days later. For biochemical experiments, DIV 14 neurons were treated and harvested via sonication in lysis buffer containing 10 mM tris (pH 8), 1 mM EDTA, and 1% SDS.

Rat cultures are typically more robust than mouse cultures (and generate more material for biochemistry) but were here used only where a direct comparison between WT and mutant cultures was not necessary (i.e., in Fig. 1, D and E, and figs. S2 and S3), and these exception are specifically indicated. Mouse was used in all other tissue culture experiments (i.e., in Figs. 3 to 5, D and E, and fig. S4), as identified by the comparative use of WT and mutant mice; mouse was also used for all biochemistry and electrophysiology in slices (see above).

### Extracellular field recordings

All recordings and analysis were performed blind to genotype. For electrical slice recording experiments, a glass micropipette (typical resistance 0.4 to 0.8 megaohm when filled with ACSF) was used to record field excitatory postsynaptic potentials (fEPSPs) from the CA1 dendritic layer in response to stimulation in the Schaffer collaterals at the CA2 to CA1 interface using a tungsten bipolar electrode. Slices were continually perfused with 30.5° ± 0.5°C ACSF at a rate of 3.5 ± 0.5 ml/min during recordings. Stimuli were delivered every 20 s, and three responses (1 min) were averaged for analysis. Data were analyzed using WinLTP software (*61*) with slope calculated as the initial rise from 10 to 60% of response peak. Input/output (I/O) curves were generated by increasing the stimulus intensity at a constant interval until a maximum response or population spike was noted to determine stimulation that elicits 40 to 70% of maximum slope. Slope of I/O curve was calculated by dividing the slope of response (millivolts per millisecond) by the fiber volley amplitude (millivolts) for the initial linear increase. Paired-pulse recordings (50-ms interpulse interval) were acquired from 40% max slope, and no differences in presynaptic facilitation were seen in mutant slices. A stable baseline was acquired for a minimum of 20 min at 70% maximum slope before lower frequency stimulation (900 pulses at 1 Hz) induced LTD or at 50% maximum slope before higher frequency stimulation (100 Hz). Slices were stimulated electrically with 900 pulses at 1 Hz or for 1 s at 100 Hz, and responses were recorded for 60 min after stimulation. Change in slope was calculated as a ratio of the average slope of the 20 min baseline (before stimulation). For NMDAR experiments, slices were treated with NMDAR antagonists (50 μM d,l-2-amino-5-phosphonovaleric acid and 10 μM MK-801, diluted in ACSF) for the entire recording session, including 10 min before I/O curve acquisition (or until 10 min baseline was achieved).

### cLTD and cLTP stimulation

cLTD was induced with 30 μM NMDA, 10 μM glycine, and 10 μM CNQX for 1 min. cLTP was induced with 100 μM glutamate and 10 μM glycine for 1 min. Both treatments were followed by washout in fresh ACSF; samples were harvested 1 min after the cLTP stimuli and 5 min after the cLTD, unless indicated otherwise.

### Slice biochemistry

For electrically stimulated biochemical experiments, CA1 mini-slices were stimulated with a tungsten mono-polar electrode to allow for a larger stimulation area of CA3-CA1 synapses. In addition, hippocampal slices were treated with cLTD and then sonicated in buffer containing 1 mM EDTA and 10 mM tris (pH 8) in 1% SDS. For all slice biochemical experiments, *n* represents the number of independent treatments that each consisted of pooled slices from at least three mice (equal number of slices from each mouse for each condition, typically two to four slices per treatment).

### Live imaging of hippocampal cultured neurons

Live imaging was performed following procedures that we have previously described (*36*). All images were acquired using an Axio Observer microscope (Carl Zeiss) fitted with a 63× Plan-Apo/1.4 numerical aperture objective, using 445-, 515-, 561-, and 647-nm laser excitation and a CSU-XI spinning disk confocal scan head (Yokogawa) coupled to an Evolve 512 EM-CCD camera (Photometrics). Experiments were analyzed using SlideBook 6.0 software [Intelligent Imaging Innovations (3i)]. During image acquisition, neurons were maintained at 34°C in ACSF solution containing 130 mM NaCl, 5 mM KCl, 10 mM Hepes (pH 7.4), 20 mM glucose, 2 mM CaCl_2_, and 1 mM MgCl_2_, adjusted to proper osmolarity with sucrose. After baseline imaging and cLTP or cLTD treatment, neurons were imaged 1 min (cLTP) or 5 min (cLTD) later. Tertiary dendrites from pyramidal spiny neurons were selected from maximum intensity projections of confocal Z stacks. To analyze synaptic CaMKIIα, the mean yellow fluorescent protein (YFP) intensity (CaMKIIα) at excitatory (PSD-95) and inhibitory (gephyrin) synapses was quantified. PSD-95 and gephyrin threshold masks were defined using the mean intensity of mCh or mTurquois plus 2 SDs. Synaptic CaMKIIα was then calculated using the mean YFP intensity at PSD-95 or gephyrin puncta masks divided by the mean intensity of a line drawn in the dendritic shaft. Changes in CaMKIIα synaptic accumulation were determined by dividing the net change in YFP at PSD-95 or gephyrin puncta-to-shaft ratio by the prestimulation YFP puncta-to-shaft ratio.

### Immunocytochemistry

Cultured hippocampal neurons were allowed to recover for 5 min after cLTP and 20 min after cLTD, then fixed in 4% paraformaldehyde and 4% sucrose in PBS for 15 min, and washed three times for 10 min with PBS. Nonpermeabilized cells were blocked in 5% BSA in PBS at room temperature for 1 hour and surface-stained with anti–GABA_A_R α1 (1:2000, Synaptic Systems) in 5% BSA in PBS overnight at 4°C. Cells were then washed three times for 10 min with PBS and incubated with Alexa Fluor 647–labeled secondary antibodies (1:500, Thermo Fisher Scientific) for 1 to 2 hours at 25°C. After washing four times for 10 min with PBS, coverslips were embedded using ProLong gold anti-fade reagent (Thermo Fisher Scientific) for confocal imaging.

### Surface biotinylation

Surface biotinylation was performed as described previously with slight modifications (*36*, *62*). CA1 mini-slices or cultured neurons were treated with cLTD, allowed to recover for 10 min, and then incubated in ACSF containing EZ-Link-Sulfo-NHS-LC-Biotin (1 mg/ml, Thermo Fisher Scientific) for 10 min at room temperature. Neurons or slices were then rinsed three times in ACSF and 0.1% BSA and sonicated in warm precipitation buffer (PB) containing 5 mM EDTA, 5 mM EGTA, 100 mM NaCl, phosphatase inhibitors [10 mM NaPO_4_, 1 mM Na_3_VO_4_, 10 mM sodium pyrophosphate, 50 mM NaF (Sodium Fluoride)], and protease inhibitors (Roche cOmplete cocktail). After clearing lysates by centrifugation (18,000*g*) for 20 min, 10% of each sample was reserved for total input quantification. The remaining sample was combined with 30 μl of NeutrAvidin agarose beads (Thermo Fisher Scientific) and incubated overnight at 4°C for pull-down of biotinylated proteins. The beads were then washed two times each with PB containing 0.1% Triton X-100, 0.1% Triton X-100 and 600 mM NaCl, and PB alone. Sample buffer was added to the beads and samples were heated at 90°C for 5 min before SDS–polyacrylamide gel electrophoresis (PAGE). Immunoblotting was performed with GluA1 (1:2000, Millipore), GABA_A_R α1 (1:1000, Synaptic Systems), and β-tubulin (1:5000, Millipore) primary antibodies. Blots were quantified by comparing biotinylated protein to total protein, after normalizing to β-tubulin loading control.

### Protein purification

All purified proteins and cell extracts were stored at −80°C. Recombinant CaMKII was purified from a baculovirus/Sf9 cell expression system, as previously described (*63*). Cells were pelleted and lysed in Brickey buffer containing 10 mM tris (pH 7.5), 1 mM EDTA, 1 mM EGTA, 1 mM β-mercaptoethanol, 2.5% betaine, and protease inhibitor (Roche cOmplete). Lysates were cleared by ultracentrifugation at 100,000*g* at 4°C for 30 min. The supernatant was loaded onto a phosphor-cellulose column, washed three times in elution buffer containing 50 mM Pipes (pH 7.0); 1 mM EGTA; 1 mM BME (Beta-Mercaptoethanol); and 100, 180, or 500 mM NaCl. Protein eluted during these washes was diluted to a final concentration of 25 mM Pipes (pH 7.0), 100 mM NaCl, 1 mM CaCl_2_, and 10% glycerol, before being incubated with CaM-Sepharose beads for 1 hour at 4°C. Beads were washed three times in equilibration buffer containing 25 mM Pipes (pH 7.0), 500 mM NaCl, 1 mM CaCl_2_, and 10% glycerol. Purified CaMKII was then eluted in Ca^2+^-free buffer containing 25 mM Pipes (pH 7.0), 400 mM NaCl, 1 mM EGTA, and 10% glycerol. To study specific CaMKII variants in vitro, HEK 293 cells were transfected with 12 μg of total cDNA/10-cm plate using Ca_2_PO_4_. To express heteromeric CaMKII, two constructs were transfected simultaneously using equal amounts of cDNA. After 48 hours, cells were harvested on ice in PBS, pelleted at 1000*g*, and homogenized in buffer containing 50 mM Pipes (pH 7.0), 1 mM EGTA, 1 mM dithiothreitol, 500 mM NaCl, protease inhibitors (Roche cOmplete), and 2 μM microcystin-LR. After clearing by centrifugation at 20,000*g* for 20 min at 4°C, CaMKII concentration was determined by SDS-PAGE and immunoblot with a purified CaMKII standard. Recombinant CaM was purified from BL21 bacteria using differential ammonium sulfate precipitation, as previously described (*64*). Transformed cells were grown until optical density at 600 nm (OD_600_) = ~0.6 before expression was induced by 1 mM isopropyl-β-d-thiogalactopyranoside (IPTG) for 3 hours. Cells were then pelleted at 2500*g* and resuspended in resuspension buffer containing 20 mM tris (pH 7.55), 150 mM NaCl, 1 mM EDTA, 0.1% Tween 20, lysozyme (1 mg/ml), ribonuclease A (10 μg/ml), deoxyribonuclease I (20 μg/ml), and protease inhibitors (Roche cOmplete). Cell suspensions were freeze-thawed, sonicated, and cleared by ultracentrifugation at 100,000*g* for 1 hour. Ammonium sulfate (3 M) was added to the supernatant, which was then centrifuged at 12,000*g* for 10 min. An additional 2 M total ammonium sulfate was added to the supernatant, which was then centrifuged at 12,000*g* for 10 min. The pellet was dissolved in 200 to 400 ml of Buffer P [50 mM tris (pH 7.5), 1 mM EDTA, and 200 mM ammonium sulfate] and then loaded onto a phenyl-Sepharose column equilibrated in Buffer P. CaCl_2_ (2.5 mM) was added to the flow-through, which was then applied to another phenyl-Sepharose column equilibrated in Buffer P and 2.5 mM CaCl_2_. After washing, protein was eluted in Ca^2+^-free buffer containing 50 mM tris (pH 7.5), 2.5 mM EGTA, and 1 M NaCl. The eluent was desalted by a gel filtration desalting column using 50 mM Mops (pH 7.0).

Glutathione *S*-transferase (GST)–fusion proteins with cytoplasmic GluA1 loop1 and C-tail were purified from BL21 bacteria, as previously described (*24*). Transformed cells were grown until OD_600_ = ~0.6 before expression was induced by 1 mM IPTG for 3 hours. Cells were then pelleted at 2500*g* and resuspended in resuspension buffer (described above). Cell suspensions were freeze-thawed, sonicated, and cleared by 10,000*g* centrifugation. The supernatant containing GST-fusion protein was batch purified with Glutathione Sepharose 4B (GE Healthcare), washed three times with TBS, and eluted with 100 mM reduced glutathione in 200 mM tris (pH 9.0). Glutathione was removed by dialysis against 2 liters of 50 mM tris (pH 7.6) and 300 mM NaCl, twice for 2 hours.

### In vitro phosphorylation assays

Kinase reactions were performed with purified CaMKII (from Sf9 cells) or overexpressed CaMKII (HEK 293 cell lysate) in Buffer A containing 50 mM Pipes (pH 7.1), 10 mM MgCl_2_, 1 mM adenosine triphosphate (ATP). Purified CaMKII (100 nM kinase subunits) was stimulated at 30°C in Buffer A with additional (i) 2 mM CaCl_2_ and 0.01 to 3 μM CaM or (ii) 0.3 versus 20 μM CaCl_2_ and 1 μM CaM. For selective autophosphorylation of T286, CaMKII (250 nM kinase subunits) was stimulated on ice for 10 min in Buffer A with additional 2 mM CaCl_2_ and 1 μM CaM, followed by addition of 5 mM EDTA. Then, for autophosphorylation of T305/306, diluted autonomous CaMKII (100 nM kinase subunits) was stimulated at 30°C with Buffer B [50 mM Pipes (pH 7.1), 10 mM MgCl_2_, 1 mM ATP, and 2 mM EGTA]. Basal kinase reactions were initiated with Buffer B (without prior T286 activation). In vitro GluA1 phosphorylation assays were done at 30°C with T286-autophosphorylated CaMKII (10 nM kinase subunits) and 2 μM GST fusion proteins of the cytoplasmic GluA1 loop1 (containing S567) or C-tail (containing S831), in the presence of either 2 mM Ca^2+^/1 μM CaM or EGTA. Reactions were terminated by adding sample buffer and heating at 95°C for 5 min.

### SDS-PAGE and immunoblot

Protein content was determined using the Pierce BCA protein assay (Thermo Fisher Scientific). Four to 10 μg of total protein was resolved by SDS-PAGE on 8 or 10% polyacrylamide gels and transferred to polyvinylidene fluoride membrane at 24 V for 1 to 2 hours at 4°C in transfer buffer containing 12 to 15% MeOH, 25 mM tris, 192 mM glycine (pH 8.3). All membranes were blocked in 5% nonfat dried milk in TBS [20 mM tris (pH 7.4) and 150 mM NaCl] with 0.1% Tween 20 (TBS-T) for 1 hour at room temperature before primary antibody incubation for 2 hours at room temperature or overnight at 4°C. Blots were then washed three times for 10 min in TBS-T, incubated in secondary antibody (1:6000 to 10,000) for 1 hour at room temperature, washed three times for 10 min in TBS-T. Immunoreactive signal was visualized by chemiluminescence (SuperSignal West Femto, Thermo Fisher Scientific) using the Chemi-Imager 4400 system (Alpha Innotech). Densitometry analysis was performed in AlphaEaseFC (Alpha Innotech) or ImageJ (NIH) software as follows: Immunoreactive bands were outlined and light intensity per area was measured. Background intensity below each band was subtracted. Phospho-signal was normalized to total protein. The relative immunodetection value was normalized as a percent of the average of all control conditions for the same blot, which was set at a value of one to allow comparison between multiple experiments.

### Quantification and statistical analysis

All data are shown as means ± SEM. Statistical significance and sample size (*n*) are indicated in the figure legends. Sample sizes for all imaging experiments indicate the number of neurons per condition. Sample sizes for electrically induced LTP and LTD experiments indicate one CA1 hippocampal mini-slice per condition. Sample sizes for chemically induced LTP and LTD experiments are either one cultured neuron well per sample or two slices per sample, using at least three independent neuronal cultures and at least three mice per dataset, unless indicated otherwise (i.e., in fig. S1A, where CA1 mini-slices from two mice were used). The analysis was performed following procedures that we have previously described (*36*). Data from the imaging experiments were obtained and quantified using SlideBook 6.0 software (3i) and analyzed using Prism (GraphPad) software. All data met parametric conditions, as evaluated by a Shapiro-Wilk test for normal distribution and a Brown-Forsythe test (three or more groups) or an *F* test (two groups) to determine equal variance. Comparisons between two groups were analyzed using unpaired, two-tailed Student’s *t* tests. Comparisons between pre- and posttreatment images at the same synapse type from the same neurons were analyzed using paired, two-tailed Student’s *t* tests. Comparisons between three or more groups were done by one-way analysis of variance (ANOVA) with Tukey’s post hoc test. Comparisons between three or more groups with two independent variables were assessed by two-way ANOVA with Bonferroni post hoc test to determine whether there is an interaction and/or main effect between the variables.

## SUPPLEMENTARY MATERIALS

Supplementary material for this article is available at http://advances.sciencemag.org/cgi/content/full/7/16/eabe2300/DC1

## Appendix

View/request a protocol for this paper from *Bio-protocol*.
